# *Mycobacterium fortuitum* skin infections after subcutaneous injections with Vietnamese traditional medicine: a case report

**DOI:** 10.1186/s12879-014-0550-z

**Published:** 2014-11-11

**Authors:** Nguyen Phu Huong Lan, Marion-Eliëtte Kolader, Nguyen Van Dung, James I Campbell, Nguyen thi Tham, Nguyen Van Vinh Chau, H Rogier van Doorn, Dien Hoa Le

**Affiliations:** The Hospital for Tropical Diseases, Ho Chi Minh, Vietnam; Oxford University Clinical Research Unit, Wellcome Trust Major Overseas Programme, 764 Vo Van Kiet, Quan 5, Ho Chi Minh, Vietnam; University of Edinburgh, Scotland, UK; Academic Medical Center Amsterdam, Amsterdam, The Netherlands; Centre for Tropical Medicine, Nuffield Department of Medicine, University of Oxford, Oxford, UK

**Keywords:** Rapidly growing mycobacteria, Mycobacterium fortuitum, Skin infection, Subcutaneous injection, Traditional medicine

## Abstract

**Background:**

Iatrogenic skin and soft tissue infections by rapidly growing mycobacteria are described with increasing frequency, especially among immunocompromised patients.

**Case presentation:**

Here, we present an immunocompetent patient with extensive *Mycobacterium fortuitum* skin and soft tissue infections after subcutaneous injections to relieve joint pains by a Vietnamese traditional medicine practitioner. Moreover, we present dilemmas faced in less resourceful settings, influencing patient management.

**Conclusion:**

This case illustrates the pathogenic potential of rapid growing mycobacteria in medical or non-medical skin penetrating procedures, their world-wide distribution and demonstrates the dilemmas faced in settings with fewer resources.

**Electronic supplementary material:**

The online version of this article (doi:10.1186/s12879-014-0550-z) contains supplementary material, which is available to authorized users.

## Background

*Mycobacterium fortuitum* is a member of the group of non-pigmented Rapidly Growing Mycobacteria (RGM), and is found ubiquitously in nature (soil, dust, and in tap water in biofilms), over a wide geographical area. Infections with non-tuberculous mycobacteria have been described increasingly, especially in immunocompromised patients and as iatrogenic infections in immunocompetent patients, causing a variety of local and disseminated disease. RGM in particular can cause local skin and soft tissue infections (SSTI) and have also been described in outbreak and pseudo-outbreak settings involving infections after surgery or other invasive procedures [1]-[3]. Here, we describe a case from the Hospital of Tropical Diseases in Ho Chi Minh City, Vietnam of *M. fortuitum* infection of the skin and soft tissues covering several joints after injection of traditional Vietnamese medicine to relieve joint pain. We discuss the diagnostic process and treatment for this patient in a setting with fewer resources.

## Case presentation

**Figure 1:**
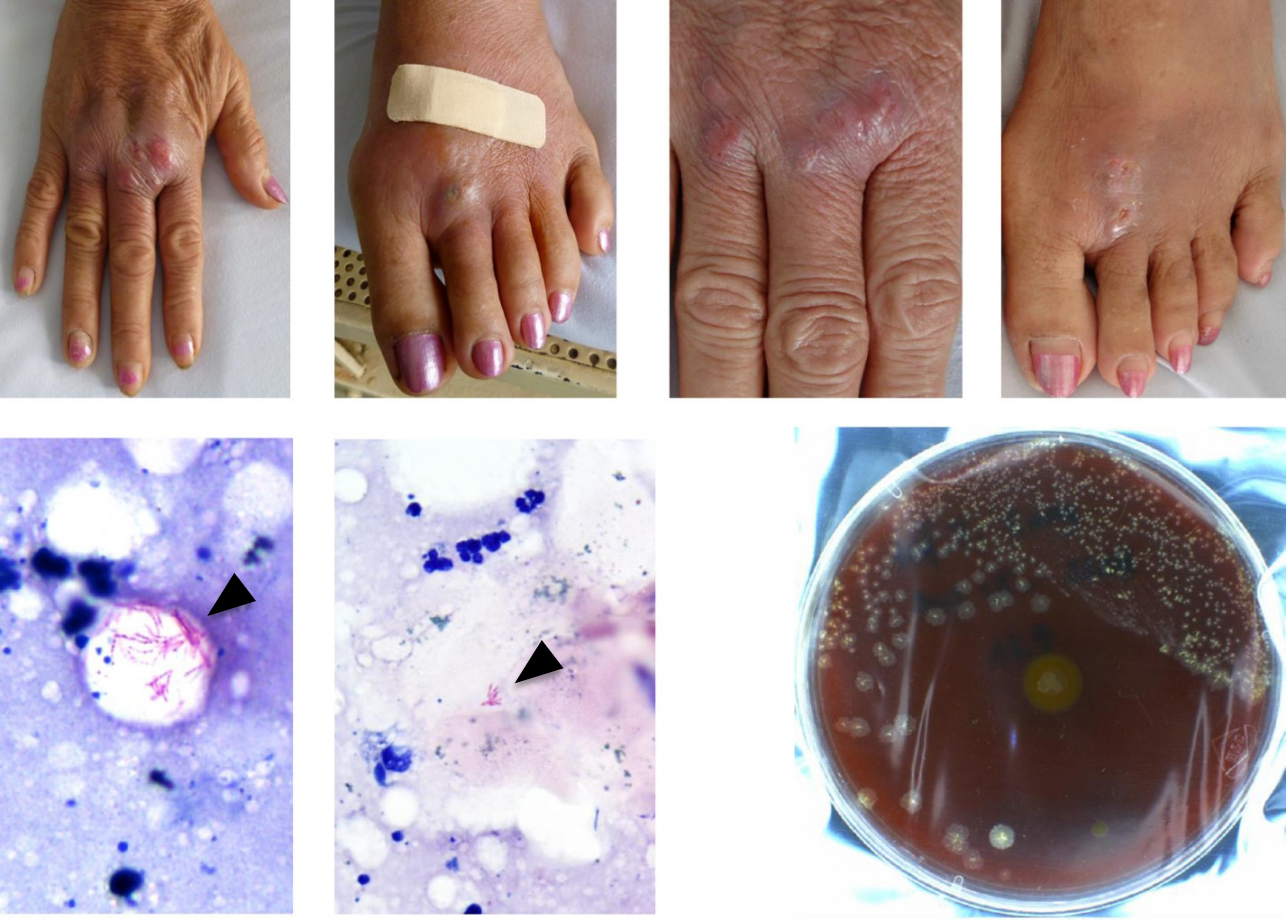
**Top, left to right: pretreatment pictures of hands and feet at day 4 and during treatment at day 67, respectively.** Bottom, left to right: two pictures of a Ziehl-Neelsen stain (1000X) of aspirated pus from the abscess on dorsal side of the patient’s right hand (acid-fast bacilli indicated with arrowheads), and a blood agar plate of aspirated pus showing non-pigmented dry colonies of *Mycobacterium fortuitum* after 4 days incubation (with two contaminating yellow colonies in the middle and bottom of the plate).

An immunocompetent, 61-year old female patient from Ho Chi Minh City, Vietnam, was admitted to the Hospital for Tropical Diseases with multiple painful fluctuating and non-fluctuating masses on both hands and feet (see Figure 1) and on her back. She was also subfebrile (37.8°C). Her previous history was uneventful except for mild hypertension and a hysterectomy because of a benign tumour. For the past 2 years she experienced numbness in both hands and feet, which was the reason for consulting a Vietnamese traditional medicine practitioner 15 days prior to admission. For three consecutive days, the patient received an oral preparation, as well as multiple subcutaneous injections (at the metacarpophalangeal and metatarsophalangeal joints of the hands and feet, and at the shoulder and hip joints) with a red substance, both of unknown composition. Five days post-injection, the injection sites became erythematous, painful, and swollen. She developed a fever and was treated at a local clinic with unknown (antimicrobial) drugs without clinical improvement, after which she was admitted to our hospital.

Laboratory findings showed: procalcitonin 0.083 ng/ml (reference range <0.05 ng/ml), leucocytes 14.8 • 10^3^/μl (6-10 • 10^3^/μl), neutrophiles 81.2% (reference range: 49-65.5%). Blood cultures (BactAlert; bioMérieux, France) were taken and empiric antimicrobial therapy was started with intravenous (i.v.) oxacilline to cover *Staphylococcus aureus* and group A beta-haemolytic streptococci.

A pulmonary X-ray, and X-ray imaging of hands and feet were unremarkable, and incision and drainage of the abscesses followed. Gram stains of the collected fluids showed slender beaded and fragmentally stained Gram positive rods, suggestive of mycobacteria. This was subsequently confirmed by Ziehl-Neelsen staining (see Figure 1). Because of reported sensitivity loss and the presence of nodules, leprosy was ruled out by investigation of ear lobe skin slits.

Based on microscopy results, a preliminary diagnosis of multiple mycobacterial skin and soft tissue infections (SSTI) with rapidly growing mycobacteria was made, and treatment was initiated with oral clarithromycine (500 mg/b.i.d.) and intramuscular (i.m.) amikacin (500 mg/t.i.d.).

Purulent fluid was cultured on standard culture media (blood agar and McConkey agar) for three days and yielded grey wrinkled colonies on blood agar (no growth on McConkey agar) (see Figure 1), morphologically suspect of *Mycobacterium spp.* This was confirmed by Gram and ZN staining and the isolate was later genotyped as *Mycobacterium fortuitum* by 16S DNA PCR and sequencing analysis.

Initial drug susceptibility testing (DST) by disk diffusion on Müller-Hinton agar showed the strain to be resistant to fluoroquinolones (ciprofloxacin, levofloxacin), azithromycin and tobramycin, but susceptible to amikacin, imipenem and amoxicillin-clavulanic acid. Clinical improvement during the 120-day admission was variable and slow, with subfebrile temperatures (38°C) last monitored on day 14, and the appearance of new fluctuating lesions on days 23 (hands/feet), 29 (hands/feet), 32 (right shoulder and hip), and 72 (shoulder), which were incised and drained. *M. fortuitum* was isolated again from the day-23 and day-29 samples, whilst microscopy and culture remained negative thereafter. Papules and blisters emerged at the sites of formerly drained sites on the hands/feet on day 72 and 80, and healed 5 days later; microbiological investigations were negative. From day 90 onwards, all lesions healed (see Figure 1), and no new skin and soft tissue abnormalities were observed.

Antimicrobial therapy was adjusted several times during admission based on repeated DST results and financial incentives. Empiric treatment was started with amikacin (i.m. 500 mg/t.i.d.) and oral clarithromycin (500 mg/b.i.d.). This was later changed to amikacin (i.m. 500 mg/t.i.d.), doxycycline (100 mg/b.i.d.) and trimethoprim-sulfamethoxazole (co-trimoxazole, 960 mg/b.i.d.), after culture results and pending additional DST. Imipenem, to which the isolate was susceptible but which the patient could not afford, was not given at this time. Once resistance to tetracyclines and co-trimoxazole were reported and amikacin had been administered for 14 days, therapy was adjusted to imipenem monotherapy i.v. 500 mg/t.i.d, and later (day 50) to imipenem 1 gram/q.i.d, based on international guidelines. Imipenem therapy was funded by the hospital. The patient was discharged on day 120, at which point imipenem was changed to oral amoxicillin-clavulanic acid (500/125 mg/t.i.d.), and follow-up visits transpired for 8 months. Antimicrobial treatment was stopped 4 months after discharge, while clinical improvement was observed 1 month after discharge (Table 1).

**Table 1:** **Clinical course and therapy**

Day	History	Diagnostics	Treatment
-15	Received injections		
-10	Start of symptoms		
	Treatment at local clinic		Unknown
0	Admission, drainage of lesions		
1		Acid-fast bacilli in ZN slide	Amikacin IV, clarithromycin
4		Culture positive	Amikacin IV, doxycyclin, co-trimoxazole
8		DST results	
		E-test imipenem	
		16S sequence	
14	Defervescene		
15			Imipenem IV, tid
23	New lesions on hands/feet	Acid-fast bacilli in ZN slide	
29	New lesions on hands/feet	Acid-fast bacilli in ZN slide	
32	New lesions on shoulder/hip	ZN/culture negative	
50			Imipenem IV, qid
72	New lesions on shoulder	ZN/culture negative	
	Papules on hands/feet		
	Papules on hands/feet		
90	All lesions healed		
110			
120	Discharge		Amoxicillin-clavulanic acid
			Stop after 4 months

Legends: ZN: Ziehl Neelsen; DST: Drug Susceptibility Test; IV: intravenous; tid: ter in die (three time a day); qid: quater in die (four times a day).

Our patient was not immunocompromised, as were 21/29 cases described by Lee et al., yet had extensive lesions which were spreading, even during therapy [4]. The number and location of lesions on initial presentation correlated with the received subcutaneous injections. Guevara-Patinos *et al*. describe *M. fortuitum* infection with multiple skin lesions after acupuncture. This was treated with doxycycline and ciprofloxacin for three months which led to full recovery [5]. Pai *et al*. describe a case of recurrent subcutaneous abscesses of unknown aetiology that did not respond to empiric antibiotic treatment and later was diagnosed as a *M. fortuitum* SSTI [6]. Because of side effects, this patient was eventually successfully treated with surgical excisions. The development of several new nodules/abscesses during antimicrobial therapy was worrisome in our case, as this could be indicative of failure of antimicrobial therapy due to misinterpretation of the susceptibility results, resistance development or dissemination in an ongoing extensive infection [7],[8]. The gold standard for susceptibility testing of RGM is the broth dilution method as described by the CLSI [8]. In our setting, broth dilution was unavailable. For imipenem an E-test was performed, for ciprofloxacin, levofloxacin, tobramycin, azithromycin, co-trimoxazole, amikacin and amoxicillin-clavulanic acid disk diffusion was performed on Müller-Hinton agar.

Our patient’s treatment regimen was altered on many occasions, but overall, 105 days of imipenem monotherapy was prescribed, in addition to the 14 days of amikacin therapy and 4 months of amoxicillin-clavulanic acid, for which the isolate was sensitive according to disk diffusion. After discharge and an 8 months follow-up period, she remained free of symptoms.

## Conclusion

In conclusion, *M. fortuitum* and other RGM are not very pathogenic, but can cause infections after direct inoculation into sterile sites, e.g. trauma with skin damage, use of contaminated surgical instruments or fluid for injection or after use of contaminated tap water during invasive procedures [7]. Infections with *M. fortuitum* appear to occur in younger patients who are generally not immunocompromised, and manifest more often after surgical procedures [9]. *M. fortuitum* is more sensitive to antibiotics than the related *M. abscessus* or *M. chelonae* and infections have a better recovery rate [10]. In our case, the isolate was relatively resistant by disk diffusion tests and only susceptible to amikacin, imipenem and amoxicillin-clavulanic acid. The patient clinically recovered after more than 12 weeks of antimicrobial therapy, which was continued for a total of 8 months. This case illustrates the pathogenic potential of RGM in medical or non-medical skin penetrating procedures and their world-wide distribution. Additional dilemmas faced in this setting with less resources were the limited availability of (biochemical) typing and susceptibility testing methods, of required antibiotics and of insurance coverage.

## Consent

Written informed consent was obtained from the patient for publication of this Case report and any accompanying images. A copy of the written consent is available for review by the Editor of this journal.

## Authors’ contributions

NVVC and DHL were responsible for patient treatment, NPHL, NTT, JC and HRVD participated in the described diagnostic process, NVD and MEK collected data and information and drafted the report, HRVD finalized the manuscript, all authors have seen and approved the manuscript.

## Authors’ original submitted files for images

Below are the links to the authors’ original submitted files for images. Authors’ original file for figure 1
