# Supplementary figures and images for: Mycobacterium fortuitum skin infections after subcutaneous injections with Vietnamese traditional medicine: a case report
Source: BMC Infect Dis. 2014 Nov 11;14:550. doi: 10.1186/s12879-014-0550-z (PMC4230753; doi:10.1186/s12879-014-0550-z)

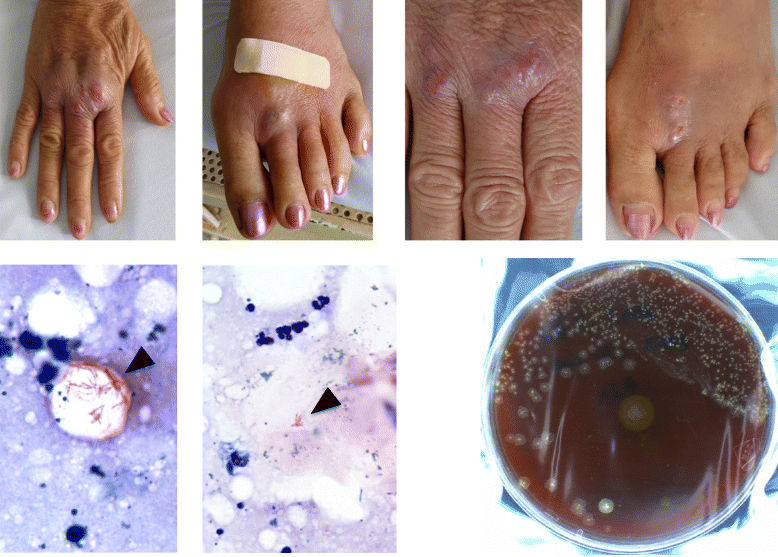

Supplement: Supplementary file 1 — Authors’ original file for figure 1 [file 12879_2014_550_MOESM1_ESM.gif]
